# Melatonin alleviates diarrhea and visceral hypersensitivity in rats with diarrhea-predominant irritable bowel syndrome by modulating of the TLR4/MyD88/NF-κB pathway

**DOI:** 10.1186/s12876-025-04203-4

**Published:** 2025-10-09

**Authors:** Zuohui Yuan, Xiaoping Yang, Xiaochun Wang, Xinlong Shi, Jia Chen, Lijun Huang, Jie Yang, Yuping Shi, Liping Zhang, Ping Mai

**Affiliations:** 1 Gansu Provincial Hospital, Lanzhou, Gansu, 730000 China; 2 The Eighth Affiliated Hospital, Southern Medical University (The First People’s Hospital of Shunde), Foshan, 528308 China

**Keywords:** Diarrhea-predominant irritable bowel syndrome, Melatonin, TLR4/MyD88/NF-κB, Intestinal microbiota

## Abstract

**Background:**

This study aimed to investigate the effects of melatonin on diarrhea and visceral hypersensitivity in rats with diarrhea-predominant irritable bowel syndrome (IBS-D) and to explore its potential mechanisms through modulation of the TLR4/MyD88/NF-κB pathway.

**Methods:**

Adult male Sprague-Dawley (SD) rats were used to establish an IBS-D model through a combination of chronic and acute stress. The rats were randomly divided into four groups: healthy control (HC), IBS-D, IBS-D + melatonin 5 mg/kg (M-L), and IBS-D + melatonin 10 mg/kg (M-H), with six rats in each group. Visceral sensitivity was assessed using the abdominal withdrawal reflex (AWR). The expression levels of tumor necrosis factor-α (TNF-α) and interleukin-6 (IL-6) in colon tissue were measured using enzyme-linked immunosorbent assay (ELISA). Western blotting and immunohistochemistry were employed to detect the expression of TLR4, MyD88, and NF-κB proteins in colon tissue. Additionally, 16 S rRNA sequencing was used to analyze the composition of the intestinal microbiota.

**Results:**

Compared to the HC group, the IBS-D group exhibited colonic inflammatory injury, increased AWR scores, elevated levels of TNF-α, IL-6, TLR4, MyD88, and NF-κB in the colon, and altered intestinal microbiota composition. Melatonin treatment reduced colonic inflammatory injury, decreased AWR scores, and lowered the levels of TNF-α, IL-6, TLR4, MyD88, and NF-κB in a dose-dependent manner. The intestinal microbiota composition in melatonin-treated groups showed a trend towards that of the HC group.

**Conclusion:**

Melatonin improved diarrhea and visceral hypersensitivity in IBS-D rats, potentially through the modulation of the TLR4/MyD88/NF-κB pathway and partial restoration of the intestinal microbiota.

## Introduction

Irritable bowel syndrome (IBS) is a common functional gastrointestinal disorder characterized by recurrent abdominal pain, bloating, and altered bowel habits [1]. The global prevalence of IBS ranges from 3 to 11% [2–4], with the diarrhea-predominant subtype (IBS-D) being particularly prevalent [5]. The etiology of IBS-D is multifactorial, involving genetic predisposition, dietary factors, gastrointestinal motility abnormalities, visceral hypersensitivity, gut-brain axis dysregulation, and alterations of intestinal microbiota [6]. Low-grade inflammation of the intestinal mucosa is considered as a key pathological feature [7]. The Toll-like receptor 4 (TLR4) plays a crucial role in IBS pathogenesis, and activation of the TLR4/MyD88/NF-κB signaling pathway is considered a major underlying mechanism [8]. Consequently, TLR4 emerges as both a potential biomarker [9] and a promising therapeutic target [8, 9] for IBS-D.

Melatonin, synthesized primarily in the pineal gland and secreted rhythmically, exhibits potent antioxidant, anti-inflammatory, and immunomodulatory properties [10, 11]. Crucially, its concentration in the gastrointestinal tract is at least 400 times higher than that in the pineal gland, highlighting significant local functions [12, 13]. Within the gut, melatonin modulates motility and sensation through serotonin pathways [14]. Clinically, it alleviates abdominal pain and improves symptom scores and quality of life in IBS patients [15, 16]. Critically, melatonin’s anti-inflammatory actions are highly relevant to IBS-D pathophysiology. It ameliorates experimental colitis [17], and prevents microbiota dysbiosis by mitigating oxidative stress and inflammation [18]. Importantly, melatonin modulates key inflammatory signaling pathways, including the TLR4/MyD88/NF-κB axis, suggesting a potential mechanism for its benefits in inflammatory gut conditions such as IBS-D.

Relevant to its anti-inflammatory potential, melatonin administration reduced TLR4-mediated inflammation and α-synuclein aggregation in a Parkinson’s disease mouse model [19], attenuated TLR4/NF-κB expression and preserved cardiac function in pulmonary hypertension [20], and improved cognitive deficits in sleep-deprived rats by inhibiting the TLR4/MyD88/NF-κB pathway and reducing neuro-inflammation [21].

Therefore, this study aimed to investigate the effects of melatonin on inflammatory markers, the TLR4/MyD88/NF-κB signaling pathway, and the intestinal microbiota composition in a rat model of IBS-D.

## Materials and methods

### Animals

Twenty-four adult male Sprague-Dawley (SD) rats (6–8 weeks old, weighing approximately 220 g) were obtained from Hubei Bainter Biotechnology Co., Ltd. The rats were housed under standard conditions with controlled temperature (22 ± 1 °C) and humidity (55 ± 5%). The animal studies were conducted in accordance with ARRIVE guidelines [22].

### Chemicals and Reagents

Melatonin was purchased from Aladdin (CAS: 73-31-4). ELISA kits for rat IL-6 and TNF-α were obtained from ELK Biotechnology. Other reagents included SDS-PAGE gel preparation kits, RIPA lysis buffer, BCA protein assay kits, and ECL chemiluminescence detection kits.

### Model development

**Fig. 1:**
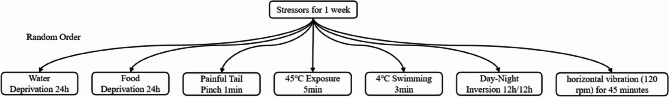
Chronic unpredictable mild stress was applied randomly every 7 days, repeated cyclically for 3 weeks

The IBS-D model was established by subjecting rats to a combination of chronic and acute stressors [23]. The CAS group was exposed to seven different stressors: (1) water deprivation for 24 h, (2) food deprivation for 24 h, (3) painful tail pinch for 1 min, (4) exposure to a 45 ℃ environment for 5 min, (5) swimming in 4 ℃ water for 3 min, (6) day and night inversion (12 h light/12 h dark), and (7) horizontal vibration (120 rpm) for 45 min. All stress protocols were applied at random every 7 days for 3 weeks, and no specific stressor was repeated on 2 consecutive days. On day 28, 1 week of rest was followed by acute restraint stress with wrapping of the shoulders, upper forelimbs and thoracic trunk for 1 h. The rats were weighed on day 0 and on day 42. Successful IBS-D induction was confirmed by statistically significant increases in fecal water content (*P* < 0.01) and AWR scores (*P* < 0.01) compared to HC prior to melatonin treatment. The details are shown in Table 1; Fig. 1.

**Table 1:** Experimental timeline

Experimental Phase	Time Period	Procedures
Baseline	D0	Body weight measurement, pre-grouping
Chronic Stress	D1-D21	7 stressors rotated randomly
Rest Period	D22-D28	No stress, environmental adaptation
Acute Stress	D28	Acute restraint stress (shoulders/forelimbs/thorax wrapped for 1 h)
Intervention	D29-D42	Intraperitoneal injection (melatonin or saline, daily at 17:00)
Endpoint	D42	Weight, fecal water content, AWR score

The animals were randomly assigned to four groups: HC group, IBS-D group, M-L group, M-H group, with 6 rats in each group. The doses of 5 mg/kg [24] and 10 mg/kg [25], considered moderate-to-high, were selected as they can reduce intestinal transit in rats [26]. Intraperitoneal injection avoids the first-pass effect, with a bioavailability of > 90% and rapid achievement of peak plasma concentration (within 15–30 min), making it suitable for acute intervention studies. The 14-day treatment course is based on covering the intestinal mucosal repair cycle (7–14 days). Upon successful model establishment, the melatonin groups received intraperitoneal injections of melatonin (5 or 10 mg/kg in 3 mL vehicle) at 17:00 each day for 14 consecutive days, while the HC and IBS-D groups were administered an equivalent volume of saline (3 mL) once daily.

### Visceral sensitivity assessment

Visceral sensitivity [27] was evaluated using the AWR in response to colorectal distension. Anesthesia was induced using a minimal amount of isoflurane at the commencement of the experiment. A catheter equipped with a latex airbag, lubricated with paraffin oil, was inserted rectally, ensuring that the airbag’s distal end was positioned 1 cm from the anal opening. The catheter was secured to the base of the rat’s tail using adhesive tape to maintain the airbag’s position. The rats were then placed in a transparent plastic cage (dimensions: 20 cm × 6 cm × 8 cm) that restricted movement. Following a 30-minute acclimatization period to ensure calmness, the catheter was connected to a syringe and a sphygmomanometer using a three-way connector. Air was gradually introduced into the airbag to induce intestinal distension, with the sphygmomanometer calibrated to increase pressure in 10-mmHg increments. Pressure values were recorded at 20, 40, 60, and 80 mmHg. Each distension lasted 20 s and was interspersed with 3-minute intervals. A scoring system was employed to quantify the degree of visceral sensitivity. To enhance the accuracy of the scoring, each level of colorectal distension was repeated three times, and the mean AWR score was used for statistical analysis.

AWR scoring criteria are as follows. 0 point: the rats remained relatively stable during colorectal distension. 1 point: slight restlessness (occasional head movements). 2 points: slight contraction of abdominal and dorsal muscles without lifting the abdomen off the ground. 3 points: stronger contractions of abdominal and dorsal muscles, resulting in the abdomen being lifted off the ground. 4 points: strong contraction of abdominal muscles, with the abdomen arching and both the abdomen and perineum elevated off the ground.

### Fecal water content

Fecal water content analysis was performed at the endpoint of melatonin treatment. The fecal water content [28] was determined using the drying method, as follows: Fresh fecal samples from rats were collected into pre-weighed weighing bottles. The wet weight of feces was recorded as W_1_. The weighing bottle containing feces was placed in a constant-temperature oven at 60 °C and dried until a constant weight was achieved. The dried feces were removed and weighed, with the weight recorded as W_2_. Fecal water content (%) was calculated using the formula: Water Content (%) = [(W_1_ – W_2_)/W_1_] × 100.

### Euthanasia protocol

Rats were deeply anesthetized using 2.5% tribromoethanol (Avertin) administered intraperitoneally at a dose of 12 mL/kg body weight. This dosage ensures rapid induction of unconsciousness and pain elimination. Loss of pedal withdrawal reflex and unresponsiveness to noxious stimuli were verified prior to euthanasia. Cervical dislocation (decapitation) was performed immediately after confirming deep anesthesia. This method aligns with AVMA guidelines for rodent euthanasia and ensures minimal distress.

### Histopathological analysis

Colon tissue samples were fixed in 4% formalin, embedded in paraffin, and sectioned at 4-µm thickness. Six sections per animal were stained with hematoxylin and eosin (H&E) for histological assessment of inflammatory damage. A scoring system was employed to quantify the degree of histopathological inflammation and villus length [29]. Inflammation scoring criteria are as follows. 0 point (normal): No neutrophil infiltration in the lamina propria, no interstitial edema. 1 point (mild): Scattered neutrophils in the lamina propria (without mucosal infiltration), mild interstitial edema. 2 points (moderate): Moderate neutrophil infiltration in the lamina propria, neutrophils present in focal areas of the mucosa, moderate interstitial edema. 3 points (severe): Dense neutrophil infiltration in the lamina propria, extending throughout the entire mucosal layer, severe interstitial edema with or without necrosis. Villus length scoring criteria are as follows. 0 point: Intact villi with normal tissue architecture. 1 point: Intact villi with separation of submucosa and lamina propria. 2 points: Mild villus sloughing, severe separation of submucosa and lamina propria, edema in submucosa and/or muscularis. 3 points: Severe villus sloughing, separation with inflamed submucosa and lamina propria, edema in submucosa and/or muscularis. 4 points: Complete villus loss with total architectural destruction and disorganization.

### Western blotting

The specific operation method of Western blotting is as follows [30]. Total protein was extracted from liquid nitrogen-preserved tissue samples. Tissues were rinsed 2–3 times with ice-cold PBS to remove blood clots or lipids, minced, and homogenized in ice-cold protein extraction reagent (10–20 × tissue volume) supplemented with 1× protease inhibitor. Additional inhibitors/stabilizers were included for low-abundance or degradation-prone proteins. The homogenate was vortexed, incubated on ice for 30 min (with intermittent pipetting), and centrifuged at 12,000 × g (4 °C, 5 min) to collect the supernatant (total protein lysate). Protein concentration was determined using a BCA Protein Assay Kit. Equal amounts of total protein (≥ 20 µg per lane) were mixed with 5 × SDS loading buffer, denatured at 95–100 °C for 5 min, and loaded onto SDS-PAGE gels. We poured separating gels (8% for TLR4/NF-κB p65; 10% for MyD88), overlaid with water, and polymerized for 45 min. Stacking gels were added, polymerized, and samples (10 µL/lane) were loaded. Electrophoresis was performed at 80 V through the stacking gel and 120 V through the separating gel. For protein transfer, PVDF membranes were pre-wetted/activated in methanol for 3 min. A transfer “sandwich” (cathode to anode: sponge → 3 filter papers → membrane → gel → 3 filter papers → sponge) was assembled bubble-free, and wet transfer was conducted at 300 mA constant current for 90 min. Membranes were blocked with 5% BSA in TBST at room temperature for 1 h. Primary antibodies diluted in blocking buffer were incubated overnight at 4 °C: TLR4 (Mouse monoclonal, Wuhan Sanying #66350-1-Ig; 1:10,000), MyD88 (Rabbit monoclonal, CST #4283; 1:10,000), NF-κB p65 (Rabbit monoclonal, CST #8242; 1:2,000), GAPDH (Rabbit monoclonal, abcam #ab181602; 1:10,000). Membranes were washed 3 times in TBST (5 min/wash) and incubated for 30 min at room temperature with HRP-conjugated secondary antibodies diluted in 5% non-fat milk/TBST: Goat Anti-Mouse IgG (ASPEN #AS1106; 1:10,000) for TLR4, and Goat Anti-Rabbit IgG (ASPEN #AS1107; 1:10,000) for MyD88, NF-κB p65, and GAPDH. After four TBST washes (5 min/wash), proteins were detected using freshly prepared ECL substrate (A: B = 1:1) and exposure to X-ray film optimized for signal intensity. Films were scanned, and band density was quantified using AlphaEaseFC software.

### Immunohistochemistry

The specific operation method of immunohistochemistry is as follows [31]. Tissue sections were deparaffinized and rehydrated through sequential incubation in xylene (15 min, twice), absolute ethanol (5 min, twice), 90% ethanol (5 min), and 75% ethanol (5 min), followed by a rinse under running tap water. For antigen retrieval, sections were rinsed in distilled water, immersed in 0.01 M citrate buffer (pH 6.0), and subjected to microwave treatment at medium-high power for 2–8 min (optimized per tissue type), then cooled to room temperature. Sections were circled with a hydrophobic barrier pen to localize reagents. After PBS rinsing, endogenous peroxidase activity was blocked using 3% H₂O₂ at room temperature for 15–30 min, followed by three 5-min PBS washes. For primary antibody incubation, sections were incubated overnight at 4 °C with rabbit anti-TLR4 primary antibody (Affinity, #AF7017) diluted 1:200 in 5% BSA. Sections were warmed to room temperature, washed in PBS (three times for 5 min each). Then, incubated with HRP-conjugated goat anti-rabbit IgG secondary antibody (Aspen, #AS-1107; diluted 1:200) at 37 °C for 30 min, followed by another three 5-min PBS washes. DAB detection was performed by applying DAB working solution and monitoring brown-yellow color development under a microscope; the reaction was stopped by rinsing with tap water. Counterstaining involved hematoxylin (3–10 min), differentiation in 1% hydrochloric acid (with rinsing), and bluing in 1% ammonia water (with rinsing); faint nuclei were optionally pre-treated with celestine blue solution (3–5 min). Sections were dehydrated through 75% ethanol (5 min), absolute ethanol (5 min, twice), and xylene (5 min, three times), then mounted with neutral balsam. Finally, imaging and analysis were conducted using an Olympus CX31 upright bright-field microscope equipped with a Q-Imaging Micro-Publisher system.

Mean optical density values and the positive areas of TLR4-positive reactants in colon tissue were quantified through microscopic image analysis. Each section was examined under a microscope connected to an image analysis system at a magnification of ×400, with five random fields of view selected for analysis. The images were processed using the IPP 6.0 (Image Pro Plus 6.0) software, which automatically calculated and averaged the integrated optical density value (IOD) of TLR4-positive expression.

Immunohistochemical quantification focused specifically on TLR4 expression due to its role as the primary receptor initiating the TLR4/MyD88/NF-κB signaling cascade. As the membrane-bound pattern recognition receptor directly interfacing with luminal pathogens/PAMPs, TLR4 exhibits distinct spatial localization within colonic tissues. This compartmentalization allows robust quantification via IOD analysis. While MyD88 and NF-κB are critical downstream effectors, their diffuse cytoplasmic/nuclear distribution limits IOD accuracy compared to Western blotting, which was employed for comprehensive pathway quantification.

### ELISA

Levels of TNF-α and IL-6 in colon tissues were measured using commercial ELISA kits following tissue homogenization: samples were rinsed in ice-cold PBS, weighed, minced, and homogenized in lysis buffer (1:9 w/v ratio) on ice. Homogenates were sonicated until clear, then centrifuged at 10,000 × g for 5 min, with supernatants stored at ≤ −20 °C. Prior to assay, supernatants were diluted 10-fold; cytokine concentrations were normalized to total protein quantified by BCA assay and expressed as pg/mg protein. Analyses used three biological replicates per group (single technical replicate per sample) with Rat IL-6 ELISA Kit (ELK Biotechnology #ELK 1158; Sensitivity: 3.3 pg/mL, Range: 7.82–500 pg/mL) and Rat TNF-α ELISA Kit (ELK Biotechnology #ELK 1396; Sensitivity: 6.1 pg/mL, Range: 15.63–1000 pg/mL), per manufacturer’s instructions.

### 16 S rRNA sequencing

The specific operation method of especially the M-H group is as follows [32, 33]. Three rats per group were randomly selected for intestinal microbiota analysis. Fecal samples from colonic contents were collected at sacrifice, stored at −80 °C, and transported on dry ice. Total microbial DNA was extracted, followed by PCR amplification targeting the V3-V4 region of the 16 S rRNA gene using primers 341 F/806R. PCR products were purified, quantified, and subjected to high-throughput sequencing on an Illumina platform via the QIIME 2 pipeline. Raw sequencing data were processed using the DADA2 algorithm for denoising, generating Amplicon Sequence Variants (ASVs) tables with single-nucleotide precision. For data analysis, α-diversity (*Shannon* index, *Chao1* richness) and β-diversity (*Bray-Curtis* distances, PCoA, PCA, NMDS, UPGMA clustering) were performed to assess microbial community structure. Taxonomic annotation was conducted against the SILVA 138 database (confidence ≥ 0.7) at phylum and genus levels. Differential abundance testing utilized Fisher’s exact test (non-replicated samples), Mann-Whitney U test (two-group comparisons with replicates), and Kruskal-Wallis test (multi-group comparisons) with *P <* 0.05 significance. Advanced analyses included LEfSe for biomarker screening, PICRUSt2 for functional prediction, BugBase for bacterial phenotype inference, and correlation network/heatmap analyses. Results were visualized via stacked bar plots, heatmaps, Venn diagrams, and phylogenetic trees, with key files stored at specified paths (e.g., ASV tables, diversity metrics, taxonomic annotations).

### Statistical analysis

For behavioral (AWR scoring) and histological (IOD of TLR4 staining) assessments, investigators were blinded to treatment assignments. All data were collected under blinded conditions. Statistical analysis was performed using SPSS 26.0 (IBM, USA). Continuous data are expressed as mean ± standard deviation (SD). After confirming normality (Shapiro-Wilk test) and homogeneity of variance (Levene’s test), between-group differences were analyzed by one-way ANOVA. Post hoc comparisons were conducted using LSD test under equal variance assumptions, while Dunnett’s T3 test was applied for unequal variances. Statistical significance was defined as *P* < 0.05.

## Results

### General observations

**Fig. 2:**
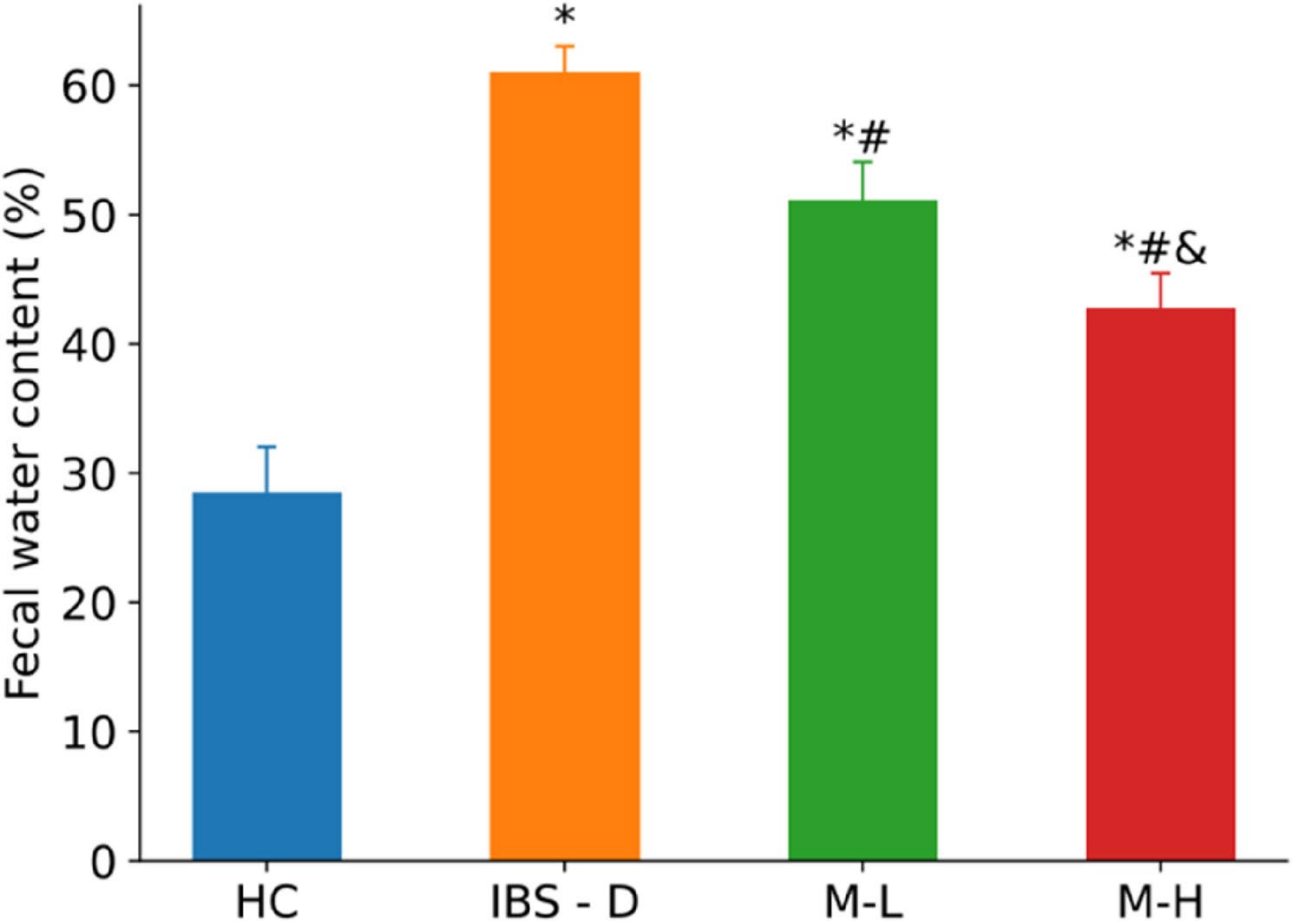
Fecal water content of rats in each group (*n* = 6). Note: Compared with the HC group, *P < 0.05. Compared with the IBS-D group, #*P* < 0.05. Compared with the M-L group, &*P* < 0.05

In the IBS-D group, the food intake of rats showed a downward trend. The feces were yellow-green, watery, and sometimes contained undigested rat food. Melatonin treatment improved these symptoms, with the M-H group showing more significant improvements. The detailed results of fecal water content are presented in Fig. 2 (IBS-D vs. HC, *P* < 0.01).

### Visceral sensitivity

**Fig. 3:**
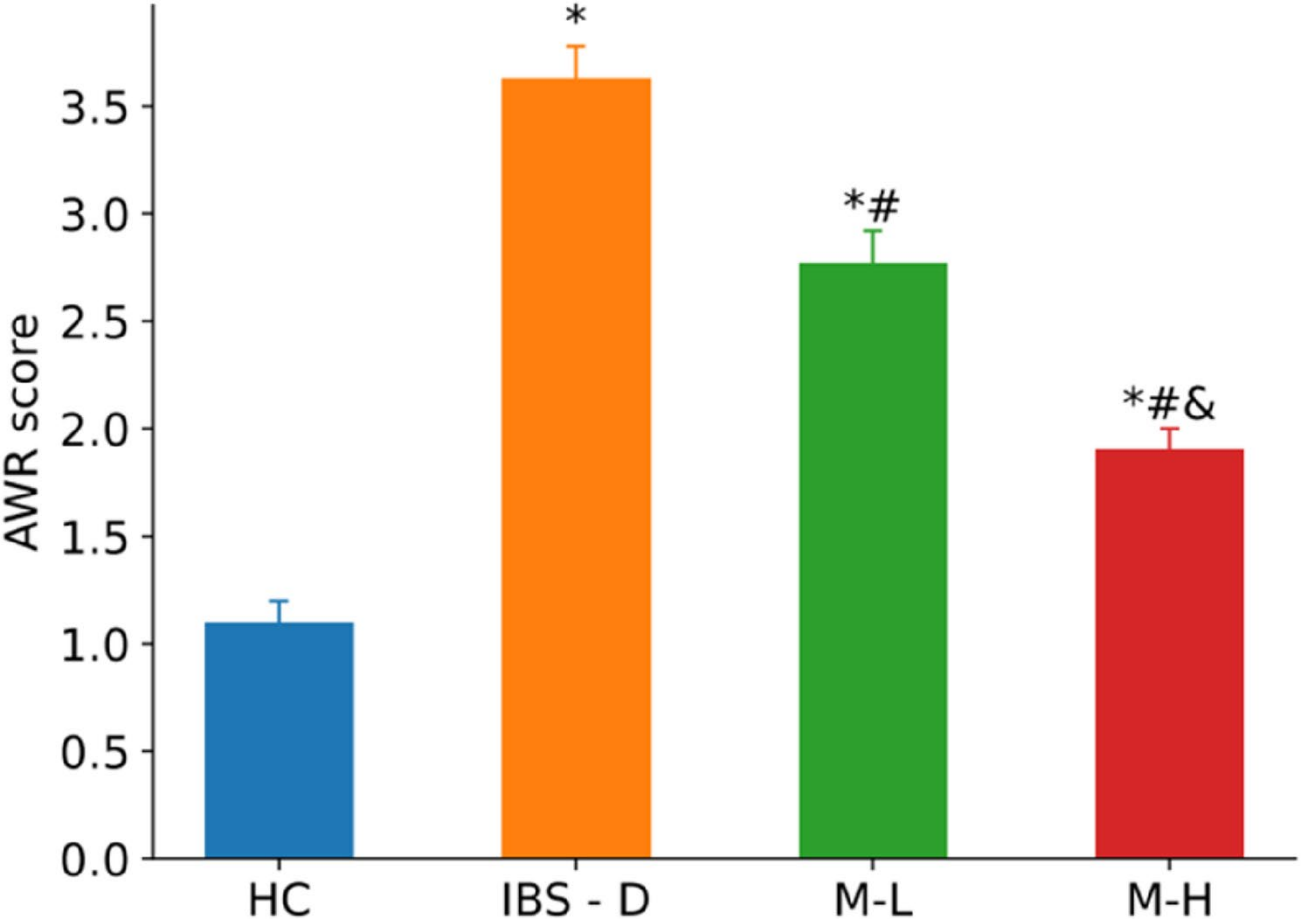
AWR scores in each group (*n* = 6). Note: Compared with the HC group, *P < 0.05. Compared with the IBS-D group, #*P* < 0.05. Compared with the M-L group, &*P* < 0.05

Melatonin treatment significantly reduced visceral hypersensitivity in IBS-D rats, as measured by AWR scores. Compared to the IBS-D group, both M-L and M-H demonstrated efficacy (*P* < 0.05), with M-H exhibiting superior dose-dependent effects versus M-L (*P* < 0.05). Despite this improvement, AWR scores in melatonin-treated groups remained elevated compared to HC (*P* < 0.05) (Fig. 3).

### Histopathological changes

**Fig. 4:**
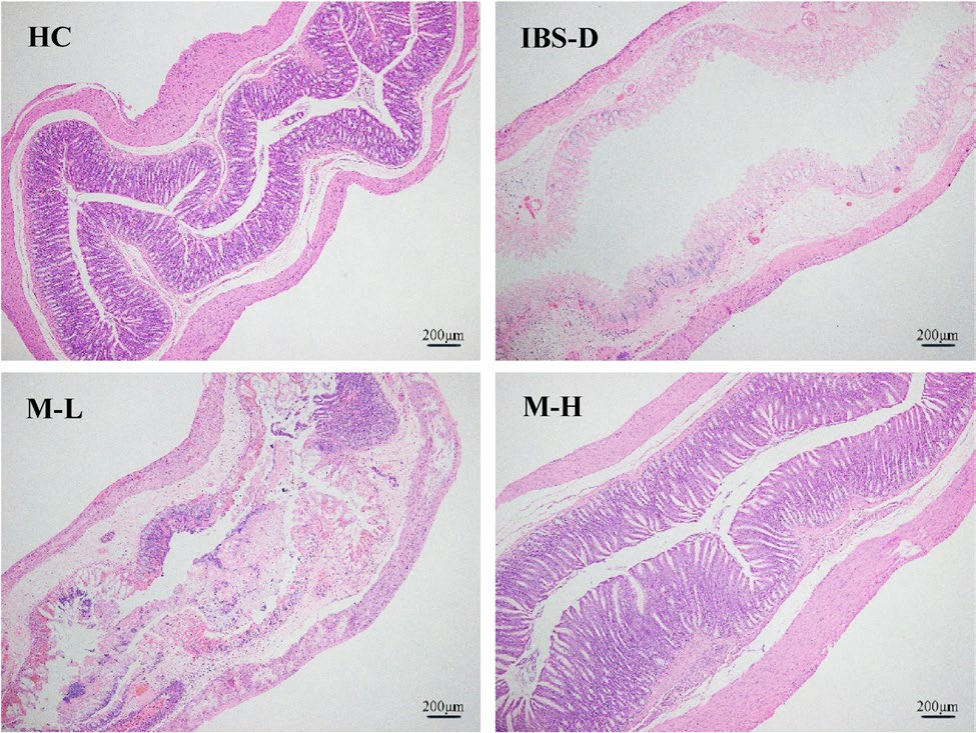
Pathological changes in the colonic mucosa of 4 groups of rats (hematoxylin-eosin, ×40)

**Fig. 5:**
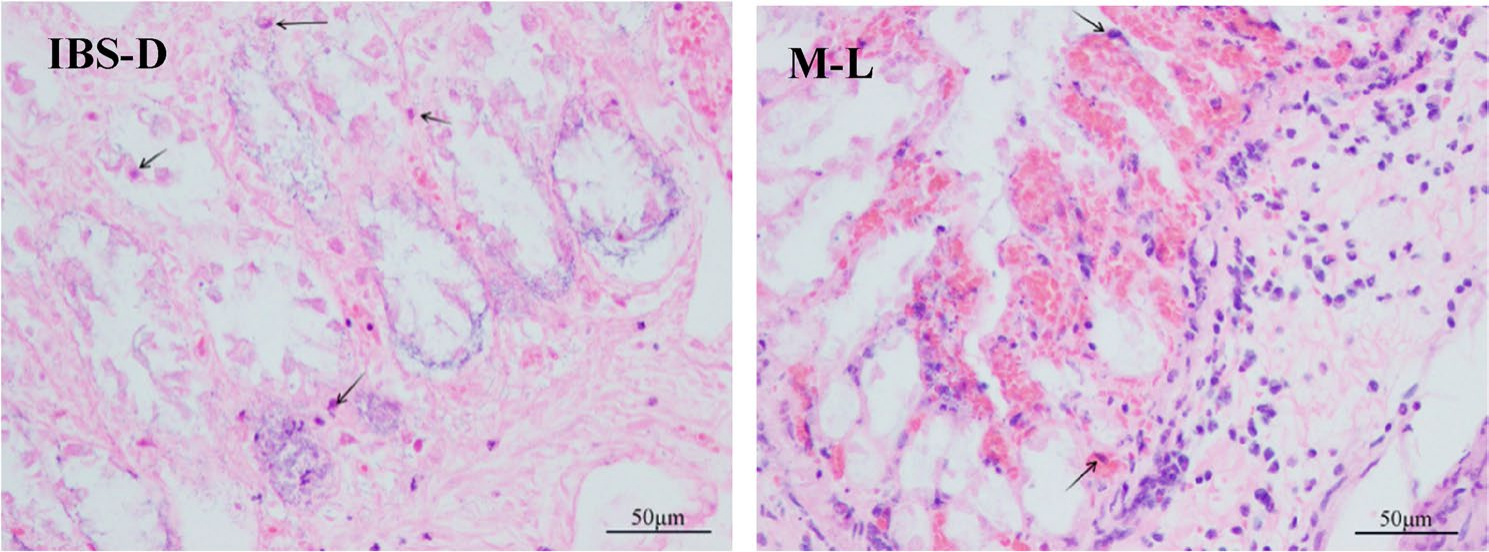
Pathological changes in the colonic mucosa of 2 groups of rats (hematoxylin-eosin, ×400). The black arrows indicate macrophage infiltration

**Fig. 6:**
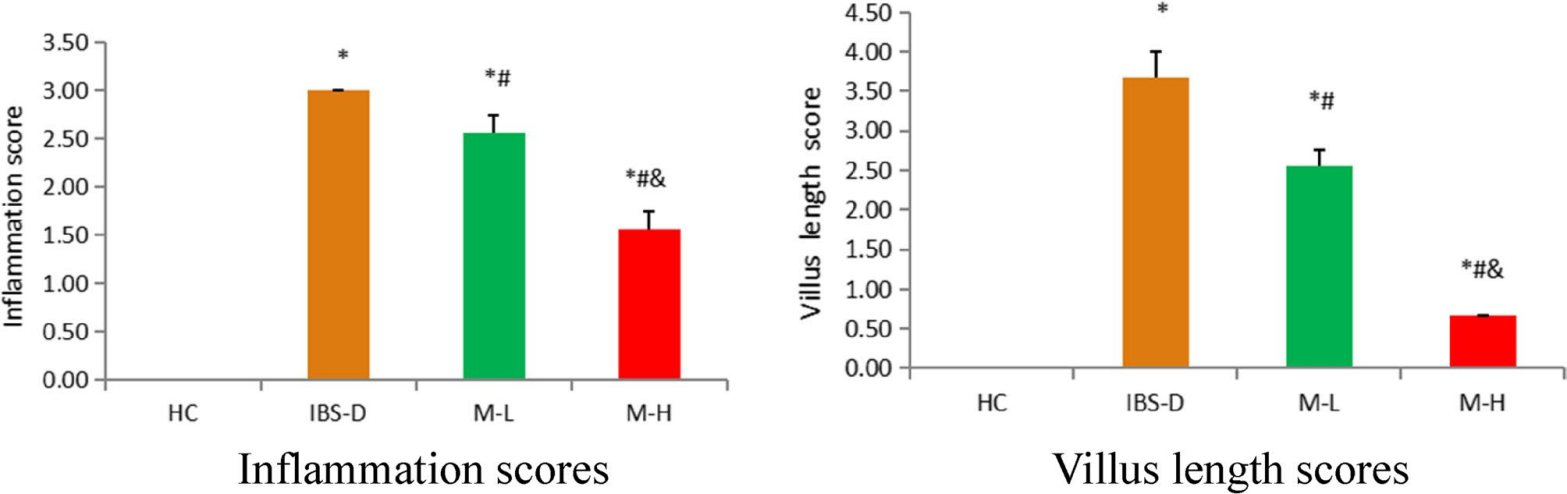
Inflammation scores and villus length scores in each group (*n* = 6). Note: Compared with the HC group, *P < 0.05. Compared with the IBS-D group, #*P* < 0.05. Compared with the M-L group, & *P*< 0.05

The IBS-D group exhibited colonic mucosal damage, accompanied by increased inflammatory cell infiltration and villi shortening. Melatonin treatment improved these changes. These results are illustrated in Figs. 4, 5 and 6.

### Inflammatory cytokines

**Fig. 7:**
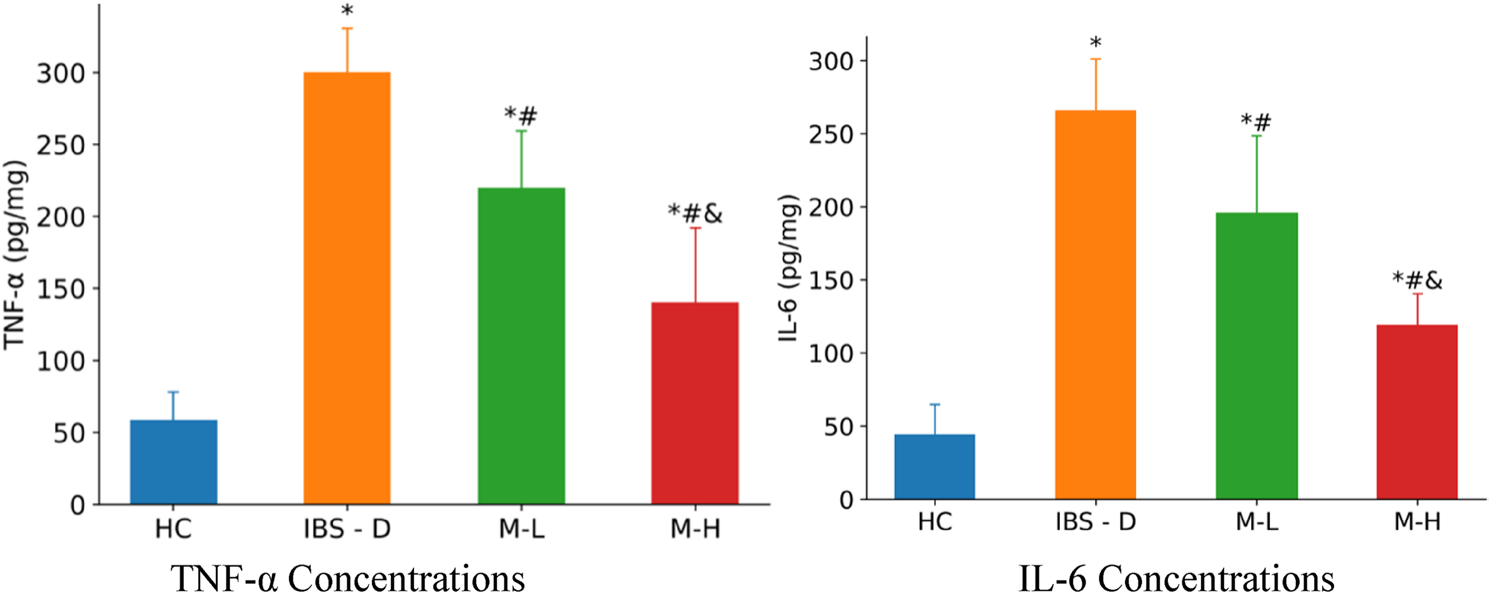
Levels of TNF-α and IL-6 in each group (*n* = 6). Note: Compared with the HC group, ^*^P < 0.05. Compared with the IBS-D group, ^#^*P* < 0.05. Compared with the M-L group, ^&^*P* < 0.05

Pro-inflammatory cytokines TNF-α and IL-6 were markedly elevated in IBS-D rats (*P* < 0.01 compared to HC). Melatonin dose-dependently suppressed these elevations, with M-H achieving significantly greater reductions than M-L (*P* < 0.05). Despite this suppression, cytokine levels in M-H remained above the HC baseline level (*P* < 0.05) (Table 2; Fig. 7).

**Table 2:** Comparison of TNF-α and IL-6 levels in intestinal mucosal tissues of rats in each group (‾x ± s)

Group	*n*	TNF-α(pg/mg)	IL-6(pg/mg)
Normal control group	6	58.43 ± 19.60	43.88 ± 20.83
IBS-D group	6	299.83 ± 31.00^*^	265.92 ± 35.33^*^
Melatonin 5 mg/kg	6	219.55 ± 39.92^*#^	195.51 ± 52.96^*#^
Melatonin 10 mg/kg	6	140.13 ± 51.81^*#&^	118.67 ± 21.75^*#&^
*F*		22.956	22.262
*P*		0.000	0.000

Note: Compared with the HC group, ^*^P < 0.05. Compared with the IBS-D group, ^#^*P* < 0.05. Compared with the M-L group, ^&^*P* < 0.05

### TLR4/MyD88/NF-κB pathway

**Fig. 8:**
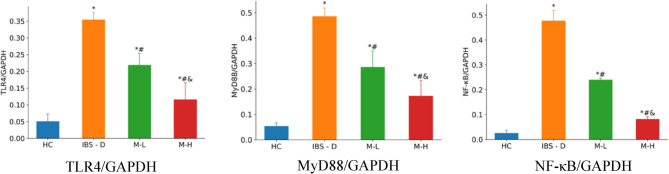
Expression of TLR4, MyD88, and NF-κB by Western Blotting in each group (n = 6). Note: Compared with the HC group, ^*^P < 0.05. Compared with the IBS-D group, ^#^*P*< 0.05. Compared with the M-L group, ^&^*P* < 0.05

**Fig. 9:**
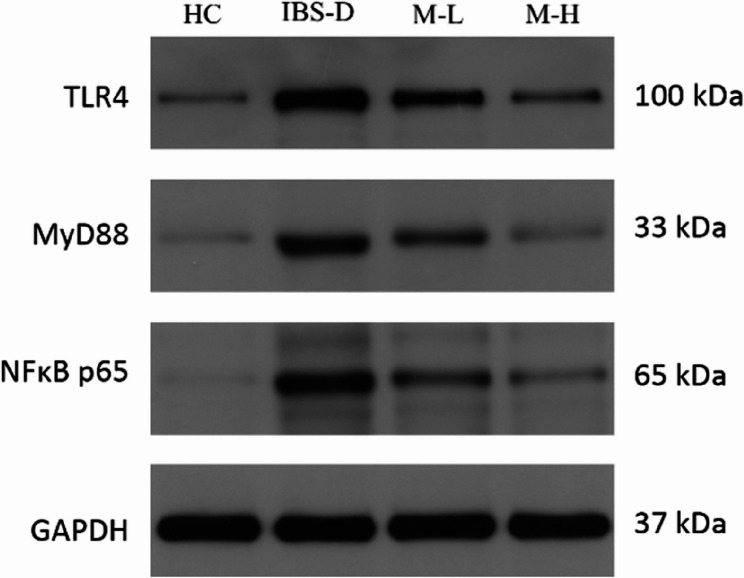
Electrophoretic gel band images of Western Blotting showing TLR4, MyD88, and NF-κB in each group

The protein expression was assessed by Western Blotting considering the entire intestinal wall (epithelium, musculature and nerve plexus). Expression of TLR4, MyD88, and NF-κB was significantly increased in the IBS-D group. Melatonin treatment reduced the expression of these proteins, with the M-H group showing more pronounced effects. The detailed results of Western Blotting are presented in Figs. 8 and 9.

**Fig. 10:**
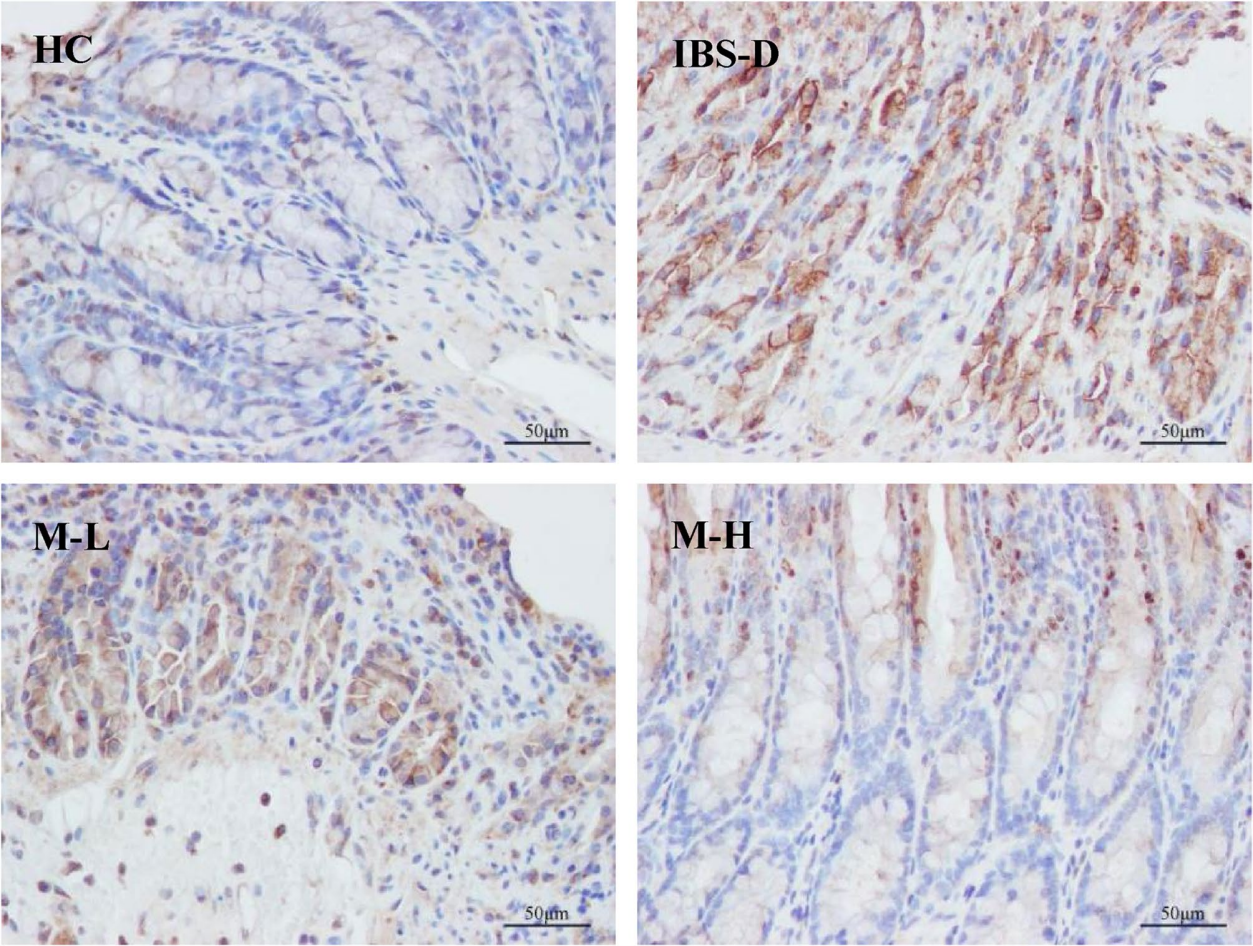
Immunohistochemical staining of TLR4 in the colonic mucosa of 4 groups of rats (×400)

**Fig. 11:**
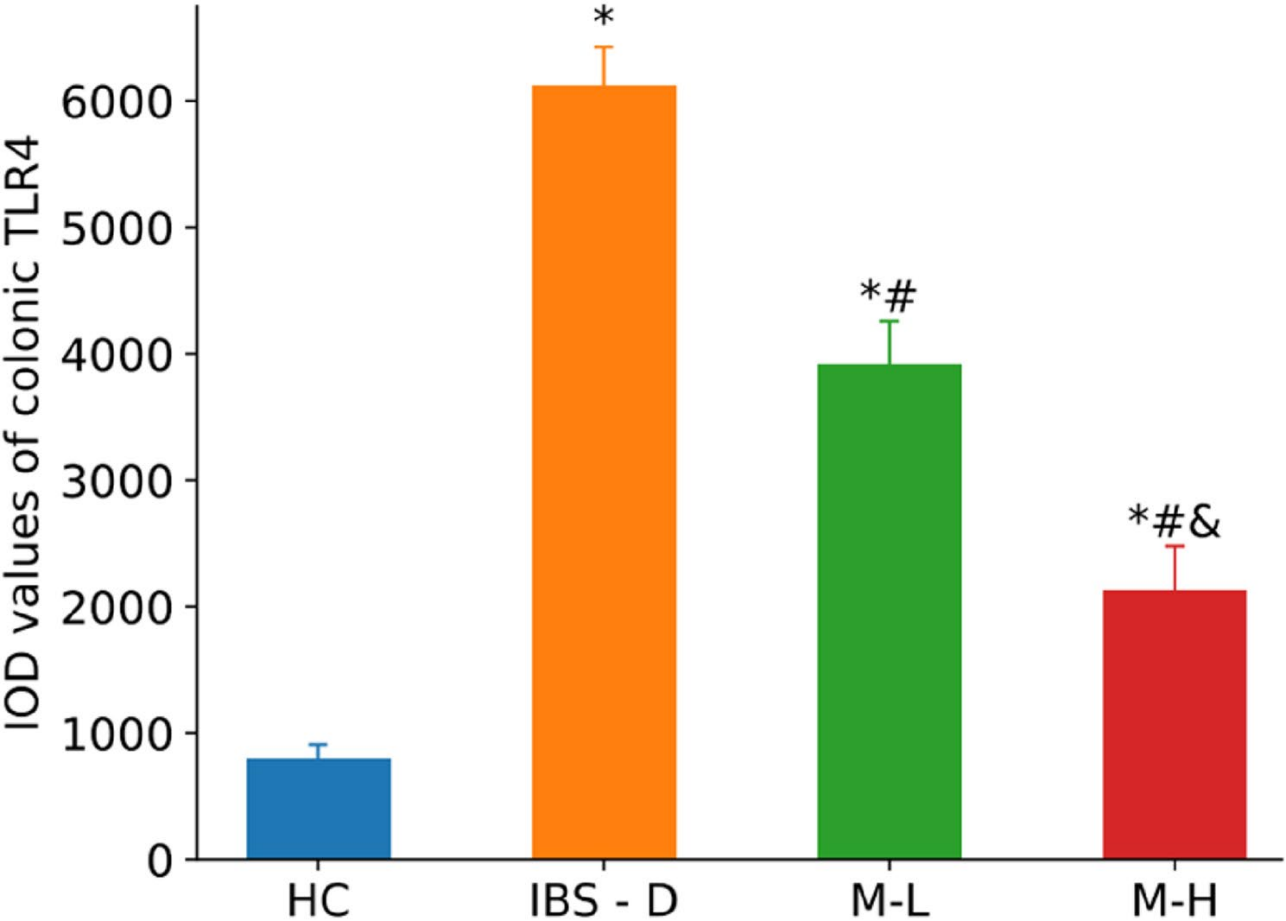
IOD of TLR4 immunohistochemical staining in each group (n = 6). Note: Compared with the HC group, ^*^P < 0.05. Compared with the IBS-D group, ^#^*P*< 0.05. Compared with the M-L group, ^&^*P* < 0.05

TLR4 was expressed in the colons of rats across all experimental groups, and was localized primarily in the cytoplasm of cells, displayed a brownish-yellow to brown coloration. The predominant sites of TLR4 expression included the cytoplasm of epithelial cells located at the apex of the mucosal layer, the cytoplasm of epithelial cells situated on the proximal side of intestinal glands adjacent to the intestinal lumen, as well as the connective tissues within the lamina propria and submucosa of the mucous membrane, and the cytoplasm of inflammatory cells. In the HC rats, colon TLR4 expression was minimal, while in IBS-D rats, it significantly increased (*P* < 0.01). In the M-L and M-H groups, TLR4 expression was notably down-regulated (*P* < 0.05), and it further decreased with increased melatonin dosage (*P* < 0.05). These findings from immunohistochemistry are illustrated in Fig. 10 and detailed in Fig. 11.

### Intestinal microbiota

**Fig. 12:**
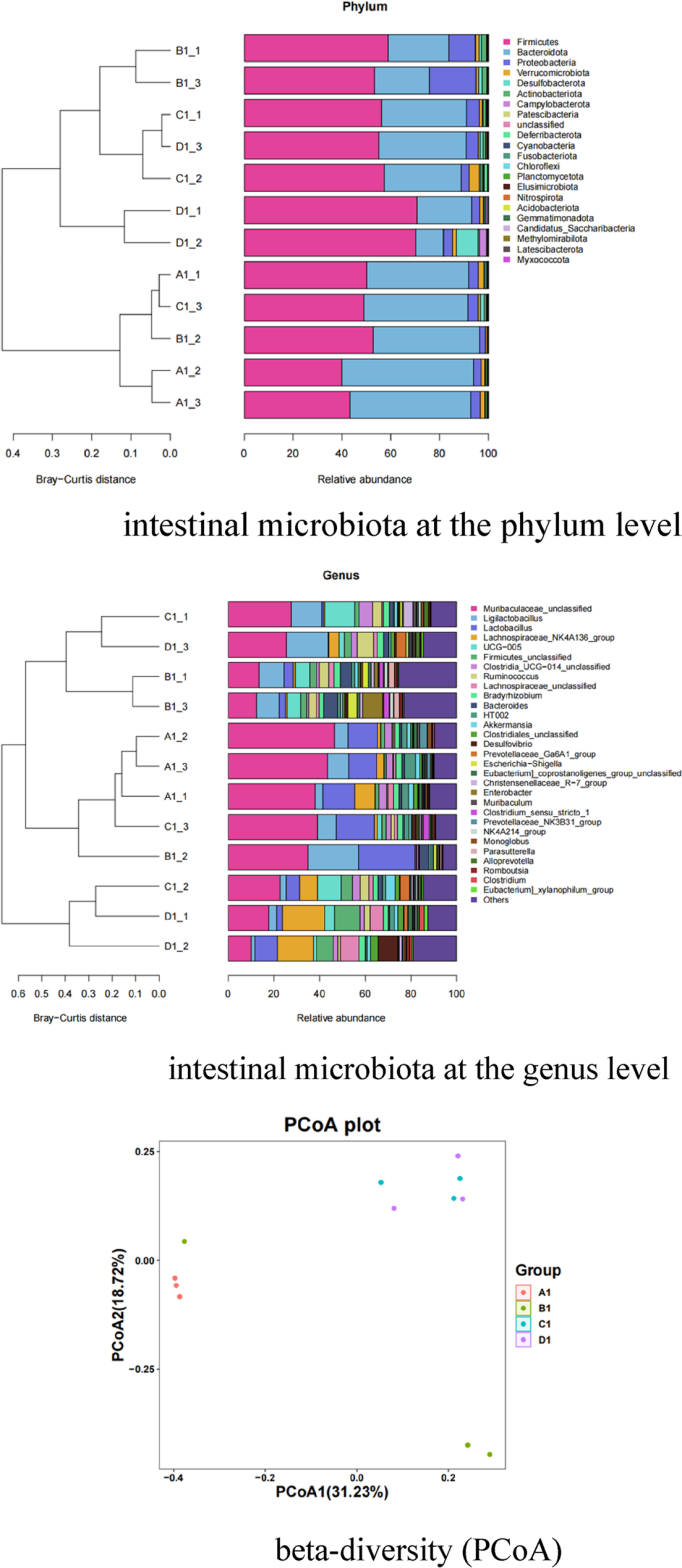
Effects of melatonin on intestinal microbiota composition in 4 groups of rats (*n* = 3). A1: the HC group; B1: the IBS-D group; C1: the M-L group; D1: the M-H group

Figure 12 demonstrates that IBS-D causes significant intestinal dysbiosis, characterized by depletion of beneficial bacteria and enrichment of pathogenic bacteria at both phylum and genus levels, along with altered community structure. Phylum Level: The IBS-D group showed a marked decrease in the relative abundance of beneficial *Firmicutes* and a substantial increase in potentially pathogenic *Proteobacteria* compared to the HC group. The *Bacteroidetes*/*Firmicutes* ratio was also significantly lower. Genus Level: The IBS-D group exhibited a significant reduction in beneficial genera (*Lactobacillus*, *Bifidobacterium*) and a pronounced increase in pathogenic genera (*Escherichia*/*Shigella*) compared to HC. Beta-Diversity: There was clear separation between the IBS-D group and the HC group, confirming a distinct and significantly altered overall microbial community structure (dysbiosis).

Melatonin treatment effectively ameliorates this dysbiosis in a dose-dependent manner, with M-H showing superior efficacy in restoring microbiota composition and structure towards a healthy state. Phylum & Genus Level: Both Melatonin treatment groups (M-L, M-H) showed a partial reversal of the dysbiosis observed in the IBS-D group. The restoration was dose-dependent, with the M-H group demonstrating a more pronounced effect: M-H treatment increased the abundance of beneficial bacteria (e.g., *Firmicutes*, *Lactobacillus*, *Bifidobacterium*) closer to HC levels. M-H treatment decreased the abundance of pathogenic bacteria (e.g., *Proteobacteria*, *Escherichia*/*Shigella*) closer to HC levels. The effect of the M-L group was evident but less robust than M-H. Beta-Diversity: Melatonin treatment, especially the M-H group, shifted the microbial community structure towards that of the HC group. The M-H group clustered significantly closer to the HC group than either the IBS-D group or the M-L group, indicating a more complete restoration of the overall microbial ecology.

## Discussion

### 1. Melatonin modulates the TLR4/MyD88/NF-κB signaling pathway

This study demonstrates that melatonin administration (5–10 mg/kg) dose-dependently attenuates TLR4/MyD88/NF-κB pathway activation in IBS-D rats. Mechanistically, this is evidenced by significant attenuation of TLR4 membrane translocation in colonic epithelium, inhibition of MyD88/NF-κB phosphorylation events, and concomitant downregulation of pro-inflammatory cytokine expression (TNF-α, IL-6). These findings align with the fact that melatonin suppresses TLR4-mediated inflammation via MyD88-dependent signaling [19–21, 34]. Notably, TLR4 is expressed not only in immune cells but also in enteric smooth muscle and neurons, where its activation disrupts nitrergic and purinergic neurotransmission, contributing to visceral hypersensitivity [35]. In TLR4-deficient models, enteric neuropathy and motility dysfunction are ameliorated, highlighting the pathway’s dual role in inflammation and neuromodulation [36]. Our results suggest melatonin’s inhibitory effect on TLR4 signaling may concurrently alleviate mucosal inflammation and restore enteric neural function.

### 2. Mechanisms underlying visceral hypersensitivity alleviation

Our study demonstrates that melatonin dose-dependently ameliorates visceral hypersensitivity in IBS-D rats, evidenced by reduced AWR scores, a well-validated visceral pain metric [37]. Critically, this analgesic effect paralleled decreased colonic TNF-α and IL-6 levels, suggesting TLR4/NF-κB signaling inhibition as a key mechanism. We propose a dual pathway: (1) Direct neuro-immune modulation via NF-κB suppression, inhibiting mast cell degranulation [38] and TRPV1 sensitization [39], and (2) Indirect barrier restoration reducing LPS translocation and subsequent TLR4/MyD88 pathway activation [18, 40].

Notably, residual hypersensitivity in treated groups implies involvement of additional pathways beyond TLR4. Melatonin’s known regulation of serotoninergic signaling [14] and hypothalamic-pituitary-adrenal (HPA) axis activity [41] may contribute, highlighting potential brain-gut axis dysregulation in persistent symptoms. This aligns with recent evidence that melatonin may modulate gut-brain axis communication through vagal afferent pathways and central neurotransmitter systems, thereby alleviating neuro-inflammation and metabolic disorders [42].

While our data establish TLR4 inhibition as pivotal, future studies should delineate melatonin’s effects on enteric glia-neuron-immune crosstalk and evaluate combinatorial therapies targeting neuroendocrine-immune interfaces for refractory symptoms.

### 3. Structural and functional restoration of intestinal microbiota

IBS-D rats exhibited dysbiosis characterized by reduced *Firmicutes* and increased *Proteobacteria* (e.g., *Escherichia*/*Shigella*), mirroring human IBS-D profiles [43, 44]. Critically, melatonin restored microbial balance by elevating beneficial genera (*Lactobacillus*, *Bifidobacterium*) while suppressing pathogens, a shift functionally linked to improved barrier-immune outcomes. The *Lactobacillus* [45] or *Bifidobacterium bifidum* [46] enrichment enhances tight junction integrity (ZO-1/occludin), and *Bifidobacterium dentium N8* reduces luminal LPS bioavailability [47]. Mechanistically, melatonin’s anti-inflammatory actions mitigate cytokine-driven dysbiosis [18, 48], while reciprocally, microbial metabolites like short-chain fatty acids (SCFAs) (e.g., butyrate) activate host GPR43 to suppress NF-κB-mediated inflammation [49, 50]. For instance, melatonin-induced *Akkermansia* proliferation reinforces mucus barriers, thus limiting TLR4 activation by translocated LPS [51]. Notably, butyrate paradoxically exacerbates visceral hypersensitivity via TRPV1 sensitization in hypersensitive states [52], highlighting context-dependent metabolite effects. This bidirectional crosstalk, in which melatonin-modulated metabolites (SCFAs/LPS) regulate TLR4/GPR43 signaling, and immune pathways feedback to shape microbiota composition, forms a self-reinforcing health-disease loop [50, 53].

### 4. Clinical translation: therapeutic positioning and dosing

Melatonin offers distinct advantages over existing treatments. Multitarget activity: Unlike antibiotics (e.g., rifaximin) or antidiarrheals (e.g., loperamide), melatonin addresses inflammation, microbiota, and visceral sensation concurrently [15, 54]. Clinical safety: FDA-classified as a safe dietary supplement, meta-analysis shows ≥ 3 mg/day for ≥ 4 weeks reduces IBS symptoms with placebo-level adverse events [11, 54]. Dose translation: The rat dose of 5–10 mg/kg (IP) converts to approximately 3–6 mg/day in humans (60 kg), and oral administration is more convenient, aligning with clinical use [55]. A recent meta-analysis confirmed melatonin improves IBS severity with a 58% pain relief rate, superior to placebo [54]. Its chronobiotic properties may also normalize disrupted intestinal motility rhythms in IBS patients [13, 16].

### 5. Study limitations and future directions

While this study elucidates melatonin’s therapeutic potential in IBS-D, several limitations warrant caution:

Translational Constraints of Animal Models: Rat models lack human IBS-D hallmarks (female predominance, comorbid anxiety/depression), limiting extrapolation to patient heterogeneity.

Whole-tissue cytokine/protein analysis could not resolve compartment-specific contributions (e.g., epithelial TLR4 vs. neuronal MyD88).

Mechanistic and Methodological Gaps: Absence of TLR4 knockout controls or MT1/MT2 receptor antagonists precludes differentiation of receptor-mediated effects from antioxidant actions.

MyD88/NF-κB signaling was assessed solely by Western blotting (no spatial localization via IHC), and TLR4 IHC omitted primary antibody negative controls.

Key gut-brain mediators (CRH, 5-HT) and microbiota metabolites (SCFAs, LPS) were unquantified, creating a “black box” in microbiota-host crosstalk.

Experimental Design Shortfalls: Small cohort size (*n* = 6/group) risks Type II errors in subgroup analyses.

14-day treatment cannot assess long-term efficacy or microbial stability.

Behavioral assessment relied solely on AWR (without electromyographic validation) and excluded anxiety tests (e.g., open-field), overlooking neuropsychiatric dimensions.

Future studies should:

Translational Relevance: Multicenter RCTs comparing melatonin vs. rifaximin (12–24 weeks) in IBS-D subpopulations (e.g., females with anxiety).

Mechanistic Specificity: Cell-type-resolved profiling (single-cell RNA-seq) + TLR4/MT1R conditional knockouts.

Pathway Validation: Integrated IHC/flow cytometry for TLR4 spatial mapping + CRF/5-HT measurements in serum/colon.

Microbiome Function: Fecal metabolomics (SCFAs/LPS) coupled with gnotobiotic FMT studies.

Behavioral Complexity: Electromyography-validated pain assays + elevated plus maze/open-field testing.

## Conclusion

This study demonstrates that melatonin attenuates diarrhea, visceral hypersensitivity, and colonic inflammation in an experimental model of IBS-D. These effects are associated with modulation of the TLR4/MyD88/NF-κB pathway and partial restoration of the intestinal microbiota. These findings support the mechanistic rationale for considering melatonin as an adjunctive therapeutic candidate in functional bowel disorders, warranting confirmation in future clinical trials.

## Data Availability

The raw sequence data reported in this paper have been deposited in the Genome Sequence Archive (Genomics, Proteomics & Bioinformatics 2021) in National Genomics Data Center (Nucleic Acids Res 2024), China National Center for Bioinformation/Beijing Institute of Genomics, Chinese Academy of Sciences (GSA: CRA023893) that are publicly accessible at https://ngdc.cncb.ac.cn/gsa. The remaining datasets used and/or analysed during the current study are available from the corresponding author on reasonable request.

## References

[1] Grayson M. Irritable bowel syndrome. Nature. 2016;533(7603):S101. 10.1038/533S101a 27191484

[2] Black CJ, Ford AC. Global burden of irritable bowel syndrome: trends, predictions and risk factors. Nat Rev Gastroenterol Hepatol. 2020;17:473–86. 32296140 10.1038/s41575-020-0286-8

[3] Oka P, Parr H, Barberio B, et al. Global prevalence of irritable bowel syndrome according to Rome III or IV criteria: A systematic review and meta-analysis. Lancet Gastroenterol Hepatol. 2020;5:908–17. 32702295 10.1016/S2468-1253(20)30217-X

[4] Liu Y, Zhang L, Wang X, et al. Similar fecal microbiota signatures in patients with diarrhea-predominant irritable bowel syndrome and patients with depression. Clin Gastroenterol Hepatol. 2016;14(11):1602–11. 27266978 10.1016/j.cgh.2016.05.033

[5] Liu YL, Liu JS. Irritable bowel syndrome in china: A review on the epidemiology, diagnosis, and management. Chin Med J (Engl). 2021;134(12):1396–401. 34074848 10.1097/CM9.0000000000001550 PMC8213251

[6] Hadjivasilis A, Tsioutis C, Michalinos A, et al. New insights into irritable bowel syndrome: from pathophysiology to treatment. Ann Gastroenterol. 2019;32:554–64. 31700231 10.20524/aog.2019.0428 PMC6826071

[7] Matricon J, Meleine M, Gelot A, et al. Review article: associations between immune activation, intestinal permeability and the irritable bowel syndrome. Aliment Pharmacol Ther. 2012;36(11–12):1009–31. 23066886 10.1111/apt.12080

[8] Wan X, Wang L, Wang Z, et al. Toll-like receptor 4 plays a vital role in irritable bowel syndrome: a scoping review. Front Immunol. 2024;15:1490653. 39749341 10.3389/fimmu.2024.1490653 PMC11693509

[9] Kim HJ. Do Toll-like receptors play a new role as a biomarker of irritable bowel syndrome?? J Neurogastroenterol Motil. 2018;24(4):510–1. 30347933 10.5056/jnm18153 PMC6175550

[10] Brzezinski A. Melatonin in humans. N Engl J Med. 1997;336:186–95. 8988899 10.1056/NEJM199701163360306

[11] Siah KT, Wong RK, Ho KY. Melatonin for the treatment of irritable bowel syndrome. World J Gastroenterol. 2014;20(10):2492–8. 24627586 10.3748/wjg.v20.i10.2492 PMC3949259

[12] Acuna-Castroviejo D, Escames G, Venegas C, et al. Extrapineal melatonin: sources, regulation, and potential functions. Cell Mol Life Sci. 2014;71:2997–3025. 24554058 10.1007/s00018-014-1579-2 PMC11113552

[13] Chen CQ, Fichna J, Bashashati M, et al. Distribution, function and physiological role of melatonin in the lower gut. World J Gastroenterol. 2011;17(34):3888–98. 22025877 10.3748/wjg.v17.i34.3888 PMC3198018

[14] Esteban-Zubero E, López-Pingarrón L, Alatorre-Jiménez MA, et al. Melatonin’s role as a co-adjuvant treatment in colonic diseases: A review. Life Sci. 2017;170:72–81. 27919824 10.1016/j.lfs.2016.11.031

[15] Song GH, Leng PH, Gwee KA, et al. Melatonin improves abdominal pain in irritable bowel syndrome patients who have sleep disturbances: a randomised, double blind, placebo controlled study. Gut. 2005;54:1402–7. 15914575 10.1136/gut.2004.062034 PMC1774717

[16] Faghih Dinevari M, Jafarzadeh F, Jabbaripour Sarmadian A, et al. The effect of melatonin on irritable bowel syndrome patients with and without sleep disorders: a randomized double-blinded placebo-controlled trial study. BMC Gastroenterol. 2023;23(1):135. 37098505 10.1186/s12876-023-02760-0 PMC10131443

[17] Nosál’ová V, Zeman M, Cerná S, et al. Protective effect of melatonin in acetic acid induced colitis in rats. J Pineal Res. 2007;42:364–70. 17439553 10.1111/j.1600-079X.2007.00428.x

[18] Wang T, Wang Z, Cao J, et al. Melatonin prevents the dysbiosis of intestinal microbiota in sleep-restricted mice by improving oxidative stress and inhibiting inflammation. Saudi J Gastroenterol. 2022;28(3):209–17. 35259859 10.4103/sjg.sjg_110_21 PMC9212112

[19] Yildirim S, Ozkan A, Aytac G, et al. Role of melatonin in TLR4-mediated inflammatory pathway in the MTPT-induced mouse model. Neurotoxicology. 2022;88:168–77. 34808223 10.1016/j.neuro.2021.11.011

[20] Lisboa CD, Maciel de Souza JL, Gaspar CJ, et al. Melatonin effects on oxidative stress and on TLR4/NF-κB inflammatory pathway in the right ventricle of rats with pulmonary arterial hypertension. Mol Cell Endocrinol. 2024;592:112330. 39002930 10.1016/j.mce.2024.112330

[21] Yin C, Zhang M, Cheng L, et al. Melatonin modulates TLR4/MyD88/NF-κB signaling pathway to ameliorate cognitive impairment in sleep-deprived rats. Front Pharmacol. 2024;15:1430599. 39101143 10.3389/fphar.2024.1430599 PMC11294086

[22] Percie du Sert N, Hurst V, Ahluwalia A, et al. The ARRIVE guidelines 2.0: updated guidelines for reporting animal research. PLoS Biol. 2020;18(7):e3000410. 32663219 10.1371/journal.pbio.3000410 PMC7360023

[23] Li S, Fei G, Fang X, et al. Changes in enteric neurons of small intestine in a rat model of irritable bowel syndrome with diarrhea. J Neurogastroenterol Motil. 2016;22(2):310–20. 26645247 10.5056/jnm15082 PMC4819870

[24] Moretti R, Zanin A, Pansiot J, et al. Melatonin reduces excitotoxic blood-brain barrier breakdown in neonatal rats. Neuroscience. 2015;311:382–97. 26542996 10.1016/j.neuroscience.2015.10.044

[25] Liu Y, Zhang Y, Wang Z, et al. Melatonin improves the ability of spermatozoa to bind with oocytes in the mouse. Reprod Fertil Dev. 2023;35(7):445–57. 37068786 10.1071/RD23006

[26] Drago F, Macauda S, Salehi S. Small doses of melatonin increase intestinal motility in rats. Dig Dis Sci. 2002;47:1969–74. 12353839 10.1023/a:1019696006677

[27] Jin Y, Ren X, Li G, et al. Beneficial effects of rifaximin in post-infectious irritable bowel syndrome mouse model beyond gut microbiota. J Gastroenterol Hepatol. 2018;33(2):443–52. 28573746 10.1111/jgh.13841

[28] Lin M, Chen L, Xiao Y, et al. Activation of cannabinoid 2 receptor relieves colonic hypermotility in a rat model of irritable bowel syndrome. Neurogastroenterol Motil. 2019;31(6):e13555. 30793435 10.1111/nmo.13555

[29] Erben U, Loddenkemper C, Doerfel K, et al. A guide to histomorphological evaluation of intestinal inflammation in mouse models. Int J Clin Exp Pathol. 2014;7(8):4557–76. 25197329 PMC4152019

[30] Sule R, Rivera G, Gomes AV. Western blotting (immunoblotting): history, theory, uses, protocol and problems. Biotechniques. 2023;75(3):99–114. 36971113 10.2144/btn-2022-0034 PMC12303220

[31] Ramos-Vara JA. Principles and methods of immunohistochemistry. Methods Mol Biol. 2017;1641:115–28. 28748460 10.1007/978-1-4939-7172-5_5

[32] Regueira-Iglesias A, Balsa-Castro C, Blanco-Pintos T, et al. Critical review of 16S rRNA gene sequencing workflow in Microbiome studies: from primer selection to advanced data analysis. Mol Oral Microbiol. 2023;38(5):347–99. 37804481 10.1111/omi.12434

[33] Hu L, Li G, Shu Y, et al. Circadian dysregulation induces alterations of visceral sensitivity and the gut microbiota in light/dark phase shift mice. Front Microbiol. 2022;13:935919. 36177467 10.3389/fmicb.2022.935919 PMC9512646

[34] Li JG, Lin JJ, Wang ZL, et al. Melatonin attenuates inflammation of acute pulpitis subjected to dental pulp injury. Am J Transl Res. 2015;7(1):66–78. 25755829 PMC4346524

[35] Caputi V, Marsilio I, Cerantola S, et al. Toll-Like receptor 4 modulates small intestine neuromuscular function through nitrergic and purinergic pathways. Front Pharmacol. 2017;8:350. 28642706 10.3389/fphar.2017.00350 PMC5463746

[36] Faggin S, Cerantola S, Caputi V, et al. Toll-like receptor 4 deficiency ameliorates experimental ileitis and enteric neuropathy: involvement of nitrergic and 5-hydroxytryptaminergic neurotransmission. Br J Pharmacol. 2025;182(8):1803–22. 39842456 10.1111/bph.17439

[37] Zhang C, Zhou X, Zhou X. Effect of acute brief social isolation on visceral pain. J Pain Res. 2022;15:3547–53. 36394056 10.2147/JPR.S378244 PMC9657267

[38] Xi M, Zhao P, Li F, et al. MicroRNA-16 inhibits the TLR4/NF-κB pathway and maintains tight junction integrity in irritable bowel syndrome with diarrhea. J Biol Chem. 2022;298(11):102461. 36067883 10.1016/j.jbc.2022.102461 PMC9647533

[39] Kim SW, Kim S, Son M, et al. Melatonin controls microbiota in colitis by goblet cell differentiation and antimicrobial peptide production through Toll-like receptor 4 signalling. Sci Rep. 2020;10(1):2232. 32042047 10.1038/s41598-020-59314-7 PMC7010660

[40] Clemente JC, Manasson J, Scher JU. The role of the gut Microbiome in systemic inflammatory disease. BMJ. 2018;360:j5145. 29311119 10.1136/bmj.j5145 PMC6889978

[41] Li Y, Jiang H, Wang X, et al. Crosstalk between the gut and brain: importance of the fecal microbiota in patient with brain tumors. Front Cell Infect Microbiol. 2022;12:881071. 35782130 10.3389/fcimb.2022.881071 PMC9247299

[42] Lv WJ, Liu C, Yu LZ, et al. Melatonin alleviates neuroinflammation and metabolic disorder in DSS-Induced depression rats. Oxid Med Cell Longev. 2020;2020:1241894. 32802257 10.1155/2020/1241894 PMC7415091

[43] Pittayanon R, Lau JT, Yuan Y, et al. Gut microbiota in patients with irritable bowel Syndrome-A systematic review. Gastroenterology. 2019;157(1):97–108. 30940523 10.1053/j.gastro.2019.03.049

[44] Tilg H, Zmora N, Adolph TE, et al. The intestinal microbiota fuelling metabolic inflammation. Nat Rev Immunol. 2020;20(1):40–54. 31388093 10.1038/s41577-019-0198-4

[45] Miyauchi E, Morita H, Tanabe S. Lactobacillus rhamnosus alleviates intestinal barrier dysfunction in part by increasing expression of Zonula occludens-1 and myosin light-chain kinase in vivo. J Dairy Sci. 2009;92(6):2400–8. 19447972 10.3168/jds.2008-1698

[46] Al-Sadi R, Dharmaprakash V, Nighot P, et al. Bifidobacterium bifidum enhances the intestinal epithelial tight junction barrier and protects against intestinal inflammation by targeting the Toll-like Receptor-2 pathway in an NF-κB-Independent manner. Int J Mol Sci. 2021;22(15):8070. 34360835 10.3390/ijms22158070 PMC8347470

[47] Zhao L, Xie Q, Etareri Evivie S, et al. Bifidobacterium dentium N8 with potential probiotic characteristics prevents LPS-induced intestinal barrier injury by alleviating the inflammatory response and regulating the tight junction in Caco-2 cell monolayers. Food Funct. 2021;12(16):7171–84. 34269367 10.1039/d1fo01164b

[48] Zhao ZX, Yuan X, Cui YY, et al. Melatonin mitigates Oxazolone-Induced colitis in Microbiota-Dependent manner. Front Immunol. 2022;12:783806. 35116024 10.3389/fimmu.2021.783806 PMC8805729

[49] Liu B, Fan L, Wang Y, et al. Gut microbiota regulates host melatonin production through epithelial cell MyD88. Gut Microbes. 2024;16(1):2313769. 38353638 10.1080/19490976.2024.2313769 PMC10868534

[50] Shabana, Shahid SU, Irfan U, et al. Crosstalk of immunity and metabolism: interaction of toll-like receptors (TLRs) and gut microbiota. Acta Diabetol. 2025;62(8):1183–94. 10.1007/s00592-025-02532-0 40471296

[51] Bonmatí-Carrión MÁ, Rol MA. Melatonin as a mediator of the gut Microbiota-Host interaction: implications for health and disease. Antioxid (Basel). 2023;13(1):34. 10.3390/antiox13010034 PMC10812647 38247459

[52] Li Y-J, Li J, Dai C. Butyrate promotes visceral hypersensitivity in IBS model via mast cell-derived DRG neuron lincRNA-01028-PKC-TRPV1 pathway. mBio. 2024;15(8):e0153324. 38953358 10.1128/mbio.01533-24 PMC11323730

[53] Chen X, Li C, Liu H. Enhanced Recombinant protein production under special environmental stress. Front Microbiol. 2021;12:630814. 33935992 10.3389/fmicb.2021.630814 PMC8084102

[54] Chen KH, Zeng BY, Zeng BS, et al. The efficacy of exogenous melatonin supplement in ameliorating irritable bowel syndrome severity: A meta-analysis of randomized controlled trials. J Formos Med Assoc. 2023;122(3):276–85. 36257872 10.1016/j.jfma.2022.10.001

[55] Huang Q, Riviere JE. The application of allometric scaling principles to predict Pharmacokinetic parameters across species. Expert Opin Drug Metab Toxicol. 2014;10(9):1241–53. 24984569 10.1517/17425255.2014.934671

